# Exploring Retinal Blood Vessel Diameters as Biomarkers in Multiple Sclerosis

**DOI:** 10.3390/jcm11113109

**Published:** 2022-05-31

**Authors:** Dragana Drobnjak Nes, Pål Berg-Hansen, Sigrid A. de Rodez Benavent, Einar A. Høgestøl, Mona K. Beyer, Daniel A. Rinker, Nina Veiby, Mia Karabeg, Beáta Éva Petrovski, Elisabeth G. Celius, Hanne F. Harbo, Goran Petrovski

**Affiliations:** 1 Center of Eye Research, Department of Ophthalmology, Oslo University Hospital, 0450 Oslo, Norway; gagid100@hotmail.com (D.D.N.); aune_sigrid@hotmail.com (S.A.d.R.B.); nina.veiby@gmail.com (N.V.); uxgrmi@ous-hf.no (M.K.); 2 Department of Neurology, Oslo University Hospital, 0372 Oslo, Norway; uxplha@ous-hf.no (P.B.-H.); einar.august@gmail.com (E.A.H.); d.a.rinker@ous-research.no (D.A.R.); uxelgu@ous-hf.no (E.G.C.); h.c.f.harbo@medisin.uio.no (H.F.H.); 3 Department of Psychology, University of Oslo, 0373 Oslo, Norway; 4 Institute of Clinical Medicine, Faculty of Medicine, University of Oslo, 0372 Oslo, Norway; mona.beyer@lyse.net (M.K.B.); b.e.petrovski@medisin.uio.no (B.É.P.); 5 Department of Radiology and Nuclear Medicine, Oslo University Hospital, 0379 Oslo, Norway

**Keywords:** retinal vessel diameter, retinal oximetry, multiple sclerosis, fundus imaging

## Abstract

We aimed to determine whether retinal vessel diameters and retinal oxygen saturation in newly diagnosed patients with multiple sclerosis (pwMS) are different from those of a healthy population. Retinal blood vessel diameters were measured using imaging with a spectrophotometric non-invasive retinal oximeter. Twenty-three newly diagnosed untreated relapsing-remitting MS (RRMS) patients (mean age: 32.2 ± 7.5 years, age range = 18–50 years, 56.5% female) were measured and compared to 23 age- and sex-matched healthy controls (HCs) (mean age: 34.8 ± 8.1 years). Patients with Optic Neuritis were excluded. Retinal venular diameter (143.8 µm versus 157.8 µm: mean; *p* = 0.0013) and retinal arteriolar diameter (112.6 µm versus 120.6 µm: mean; *p* = 0.0089) were smaller in pwMS when compared with HCs, respectively. There was no significant difference in the oxygen saturation in retinal venules and arterioles in pwMS (mean: 60.0% and 93.7%; *p* = 0.5980) compared to HCs (mean: 59.3% and 91.5%; *p* = 0.8934), respectively. There was a significant difference in the median low contrast visual acuity (2.5% contrast) between the pwMS and the HC groups (*p* = 0.0143) Retinal arteriolar and venular diameter may have potential as objective biomarkers for MS.

## 1. Introduction

Multiple sclerosis (MS) is the most common cause of neurological disability in young adults, affecting approximately 2.8 million people worldwide, with variable progression and prognosis [1]. MS is a chronic, immune-mediated disease of the CNS that often results in visual morbidity [2,3,4]. Early features of MS may include eye motility difficulties and optic neuritis (ON) as presenting ocular sign in 20% of patients, and, as the disease progresses, inter-nuclear ophthalmoplegia [5]. Other ocular conditions, including retinal vasculitis and uveitis, have been associated with MS [6,7].

The retinal ganglion cells (RCGs) and the optic nerve are commonly affected in MS in the form of optic neuritis ON; chronic MS can cause RGC atrophy, which can occur independently from ON [4,8,9,10,11].

Retinal involvements in MS have included qualitative changes in the retinal blood vessels, such as histopathological changes remarkable of vascular inflammation in 20% of the patients with MS (pwMS) in post mortem specimens [12], as well as clinical changes remarkable of retinal phlebitis in 16% of pwMS [13].

Quantitative retinal blood vessel metrics are potential markers of MS due to a known clinical relationship between MS and retinal vessel inflammation [13,14,15], and between MS and RGC loss, which potentially decreases the metabolic demand [16,17]. Structural and physiological abnormalities have been reported in the retina in pwMS [18]. Although retinal vasculitis is common in MS, it is not known if MS is associated with quantitative retinal blood vessel abnormalities.

In MS, low-contrast letter acuity (LCLA) of 2.5% contrast level has proven to be a valuable marker of residual deficits after ON, and serving as a marker of disease progression and an outcome measure in clinical MS research [19]. As visual dysfunction is one of the most common manifestations of MS, and sensitive visual outcome measures are important in examining the effect of treatment.

Spectrophotometric non-invasive retinal oximeter imaging is a technique that generates high-resolution images of the retinal blood vessels; it can measure the oxygen saturation inside the retinal vessels, as well as the vessel diameters. Only a single pilot study exists about the relationship between MS and retinal vessel diameters, which was based on pwMS with history of ON [20].

We hypothesized that the oxygen saturation and retinal vessel diameter in newly diagnosed pwMS would be significantly different from those of HCs.

This study is an important initial step in evaluating measurements of retinal vessel structure as a possible measurable in vivo biomarker of MS, and represents a novel method for assessment of retinal blood vessel metrics in this patient population.

## 2. Materials and Methods

This is a cross sectional study on pwMS diagnosed and followed at Oslo University Hospital (OUH), Oslo, Norway. In a collaboration between the Departments of Ophthalmology and Neurology at OUH, patients enrolled in Oslo in the MultipleMS study (Horizon 2020 programme, grant agreement 733161) were also referred to an eye examination (in the period 2018–2021). The MS study was approved by the regional committee for research ethics (Ref. 2011/1846-41). Written consent was obtained from the subjects.

The patients were approached to join the study by their treating neurologist, and referred to the ophthalmology department if they consented within two weeks of diagnosis and before starting any disease-modifying therapies. Inclusion criteria were a confirmed diagnosis of MS according to the revised diagnostic McDonald criteria [21,22], as well as age 18–50 years and fluency in Norwegian. Exclusion criteria were no prior ophthalmological, neurological, or psychiatric disease, no head injury, and no substance abuse. Eyes with ON were also excluded from eye examinations. Twenty-three newly diagnosed pwMS were included and compared to twenty-three healthy individuals that were age- and sex-matched. We excluded some of the eyes (1 control, 1 eye from each of 2 different patients) due to missing data or ON.

At the time of diagnosis, all pwMS underwent an MRI of the brain and spine, a lumbar puncture, and detailed neurological examinations, including the Expanded Disability Status Score (EDSS) [23]. EDSS ranges from 0 to 10 in 0.5 unit increments that represent higher levels of disability. EDSS steps 1.0 to 4.5 refer to people with MS who are able to walk without any aid.

Full ophthalmic examination was performed by an experienced ophthalmologist, including indirect ophthalmoscopy. In addition, digital fundus imaging was performed with the fundus camera of the oximeter (Oxymap T1, Oxymapehf., 102 Reykjavik, Iceland), andretinal vessel diameter measurements and oximetry of the retinal blood vessels were performed with using the Oxymap analyses software, as explained later.

Both Corrected Distance Visual Acuity (CDVA) and low contrast letter acuity (LCLA) of 2.5% were measured by an optometrist with an Early Treatment Diabetic Study (ETDRS) visual acuity chart in both eyes and presented as logarithm of the minimum angle of resolution (logMAR) [24]. The recommended test distance of 4 m was respected. The same examination room was used for all study participants and light was lit with approximately 100 lux.

### 2.1. Oximetry Imaging and Measurements

The retinal oximetry procedure has been described previously [25,26]. The patient was positioned in front of the fundus camera of the oximeter (Oxymap T1, Oxymapehf., Reykjavik, Iceland), and images were taken from both eyes. Two or more fundus photographs centered on the optic disc were taken, and the image with the best quality was used for the analyses. Image quality higher than 6 on the scale from 0 to 10 was considered acceptable. Retinal blood vessel diametersand oxygen saturations were measured by an experienced ophthalmologist (DDN) using the Oxymap analyses software (Oxymap T1, software 2.2.1., version 5436, Reykjavik, Iceland) according to a standardized protocol (Version 21, November 2013; Oxymap Inc., Reykjavik, Iceland) [26,27].

The algorithm for vessel detection and diameter measurements utilizes a supervised classifier that classifies each pixel as belonging to a vessel or background. The algorithm recognizes the pixels in the center of each vessel, evaluates a vessel vector perpendicular to vessel direction, and calculates vessel diameter from the center to the last pixel belonging to the vessel in each direction [28]. Each pixel is approximately 9.3 μm.

Arterioles and venules wider than 8 pixels (74 µm) and longer than 50 pixels (480 µm) were measured in a peripapillary annulus within a standard grid of 1.5 to 3.0 disc diameters from the optic disc center. The right eye from each patient was measured; however, if the image quality was poor or the vessels were ungradable, the left eye was measured instead. The oxygen saturation results are influenced by the vessel diameter, but that is automatically corrected by the analysis software [27].

### 2.2. Statistics

Data analysis was performed using descriptive statistical analysis; percentage distribution, and mean and standard deviation (SD). In case of non-normality of continuous variables, median and interquartile ranges (IQR, measure of variability) were calculated. Normality of continuous variables was tested on a Q-Q-plot and by the Shapiro–Wilk and Kolmogorov–Smirnov test. When the normality assumption was satisfied, the student t-test was used to compare means of continuous and numerical variables; otherwise, the Mann–Whitney test was used. Homogeneity of variance was analyzed with Levene’s test; if the Levene’s test was not satisfied, the Welch test was used instead. Chi-square (χ^2^) test was used to test the differences of the distribution of categorical variables. Level of significance was set to *p* < 0.05.

Statistical Package for STATA (Stata version 14.0; College Station, TX, USA) was used for the statistical analysis.

## 3. Results

Some of the eyes (one control, one eye from each of two different patients) were excluded due to missing data or ON. (Table 1).

The retinal venular diameter (mean: 143.8 µm versus 157.8 µm; *p* = 0.0013) and retinal arteriolar diameter (mean: 112.6 µm versus 120.6 µm; *p* = 0.0089) were smaller in pwMS when compared with HCs, respectively (Table 2).

There was no significant difference in the oxygen saturation in retinal venules and arterioles in pwMS (mean: 60.0% and 93.7%, respectively) compared to HCs (mean: 59.3% and 91.5%, respectively) (Table 2).

There was, however, a significant difference in the median low contrast visual acuity (2.5% contrast) between the pwMS and the HC groups (*p* = 0.0143) (Table 2).

**Figure 1:**
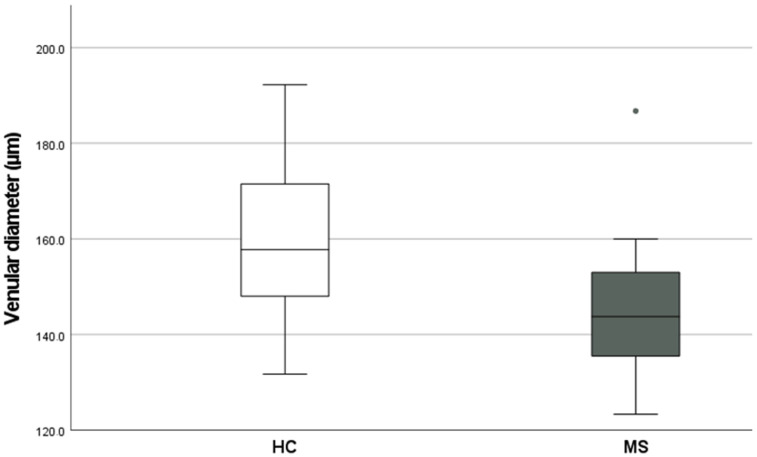
Distribution of retinal venular diameter between the healthy controls (HC) and patients with MS groups. Data presented are in the form of median (IQR: Interquartile range). HC: Healthy Controls; MS: Multiple sclerosis, *p* < 0.05.

Retinal venular diameter was wider in healthy controls (HC) compared with patients with MS groups (Figure 1). 

**Figure 2:**
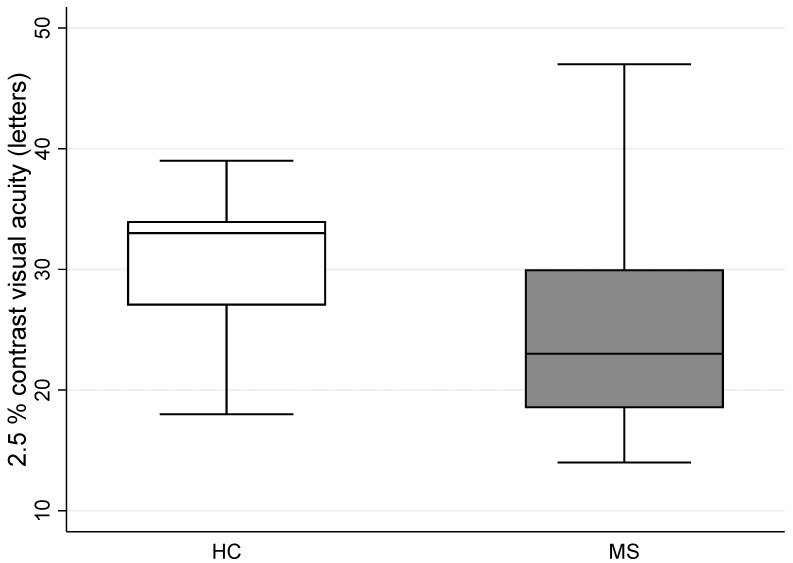
Box chart of 2.5% contrast visual acuity (letters) in the left eye between the healthy controls (HC) and MS patients’ groups. Data presented are in the form of median (IQR, Interquartile range). HC: Healthy Controls; MS: Multiple sclerosis, *p* < 0.05.

Box chart of 2.5% contrast visual acuity (letters) in the left eye between the healthy controls (HC) and MS patients’ groups is presented in Figure 2. 

A higher proportion of participants in the study were males. Time since MS diagnosis was 2 weeks. We excluded some of the eyes (one control, one eye from each of two different patients) due to missing data or ON.

## 4. Discussion

This study indicates the existence of narrower retinal vessel diameters in newly diagnosed pwMS compared with HCs. The measurement of the vessel diameter and retinal oximetry has been previously shown to be reliable and repeatable [29], with older age possibly affecting retinal oximetry parameters, which seems irrelevant to the younger population studied here.

A potential explanation for the smaller diameter of retinal vessels in MS subjects may be the presence of RGC loss, leading to lower metabolic demands on the retinal circulation. Wang et al. showed lower ON head blood flow in pwMS with history of ON, compared with both pwMS without a history of ON, and HCs, which stands in support of such a mechanism [29]. Bhaduri et al. used optical coherence tomography (OCT) to show that MS eyes had a lower total blood vessel diameter (BVD) and blood vessel number (BVN) than control eyes [18].

The relationship between the disturbances in cerebral venous outflow and neurological disorders remains an open issue that requires further studies. The high degree of comorbidity between vascular diseases and MS suggest that vascular pathology may be an important factor causing neuronal dysfunction or degeneration in MS [30,31]. There is also evidence suggesting that pwMS are more susceptible to cardiovascular risk factors than HCs, some having demonstrated regional cerebral perfusion abnormalities in these patients [20,30,31].

The decrease in blood vessel diameter has been related to qualitative changes resembling inflammatory pathology in the retinal vessels in MS. Furthermore, retinal phlebitis has been reported to correlate with MS activity, which is similar in pattern to the associations we found for retinal blood vessel diameter [18]. It could be possible that peripapillary changes represent adaptive mechanisms related to present or past phlebitis in the peripheral retina. This evidence is indirect, since the location of the qualitative pathology (in the retinal periphery) is distant from the location of our blood vessel measurements (found around the ON). However, the connected nature of the retinal blood vessels means changes in the proximal vessels are possibly associated with distal pathology. Future studies are needed to compare the clinical and imaging findings in pwMS in order to determine if retinal blood vessel diameter is associated with current or past retinal phlebitis.

There was a significant average difference in low-contrast visual acuity between the HCs and pwMS groups, while no significant difference was found between the oxygen saturation in retinal arterioles and retinal venules in the two groups. There is insufficient knowledge about newly diagnosed MS and its relation to oxygen and vascular supply parameters in the eye. One cohort study on eight pwMS with history of ON found the mean retinal venular oxygen saturation to be higher in pwMS than in HCs [20].

Overall, changes in the retinal vessel diameter, in particular smaller arterioles, have been related to cardiovascular mortality (from coronary heart disease and stroke) [32,33]. In addition, such changes have been found to contribute to the development of diabetic retinopathy, in particular, early stages, and considered to be prognostic markers of the disease [34]. Similarly, we have found that retinal venular oxygen saturation is associated with early stage non-proliferative diabetic retinopathy in type 1 diabetes patients [26].

To our knowledge, our study represents the first application of objective, in vivo ascertainment of retinal oxygenation and vessel diameter in newly diagnosed MS patients, and it confirmed differentiation of parameters measured in controls. A study on newly diagnosed MS patients and retinal oxidative/vascular risk factors has not been performed previously in a Norwegian population, and no large international studies exist either.

Our study has several limitations. It was a pilot study with low number of participants. Blood pressure (BP) was not measured at the same. Retinal venular diameter was not statistically normally distributed, probably due to the low number of participants.

Further studies are needed to confirm our findings, and to explore the implications and biological basis of the decreased blood vessel diameter and vessel density in MS.

## 5. Conclusions

In conclusion, we found smaller retinal venular and arteriolar diameters in pwMS. If confirmed by longitudinal follow-up, this may be a useful and objective biomarker for neurodegeneration in MS.

## Figures and Tables

**Table 1:** Subject demographics and study group characteristics.

	Healthy Controls	People with MS
Eyes, *n* (Patients, *n*)	45 (23)	44 (23)
Mean Age, years (SD)	34.8 (8.1)	32.2 (7.5)
Female sex, *n* (%)	13 (56.5)	13 (56.5)
Time since diagnosis, weeks (SD)	-	2 (SD)
Optic Neuritis, *n* (Patients, *n*)	0 (0)	2 (2)
EDSS, *n* (Patients, *n*): 0; 1.0; 1.5; 2,0; 2.5; 3.0	-	3; 6; 6; 3, 4, 1

EDSS: Expanded Disability Status Scale.

**Table 2:** Vessel diameters, oxygen saturation, and 2.5% contrast visual acuity in the healthy controls and people with MS.

Variable	Healthy Controls (*n* = 23)	People with MS (*n* = 23)	*p*-Value
Retinal arteriolar diameter (µm) Mean (±SD) 95% CI	120.6 (11.5) 115.4–126–0	112.6 (10.7) 108.1–11.0	0.0013
Retinal venular diameter (µm) Median, Range (IQR)	157.8 (148.0–171.5)	143.8 (123.3–186.8)	0.0089
A-V difference in % Mean (SD) 95% CI	38.3 (14.8) 31.5–45.0	31.8 (12.0) 27.0–37.0	0.0527
Arteriolar O_2_ saturation (%) Mean (±SD) 95% CI	91.5 (7.3) 88.2–95.0	93.7 (4.2) 92.0–95.5	0.8934
Venular O_2_ saturation in% Mean (±SD) 95% CI	59.3 (9.5) 55.0–64.0	60.0 (4.6) 92.0–95.5	0.5980
2.5% contrast visual acuity, number of letters Left eye Median, Range (IQR)	31 (27.0–34.0)	23 (18.5–30.0)	0.0143
High contrast visual acuity Right eye /Left eye Mean (±SD) 95% CI	0.43 (0.09) 0.38–0.47/ 0.44 (0.12) 0.38–0.50	0.35 (0.23) 0.25–0.45/ 0.43 (0.24) 0.33–0.53	0.0722 0.04022

## Data Availability

Data supporting reported results can be provided by the corresponding authors upon request.
